# Online Commentary in Primary Care and Emergency Room Settings

**DOI:** 10.1002/ams2.229

**Published:** 2016-07-19

**Authors:** John Heritage

**Affiliations:** ^1^ Department of Sociology University of California Los Angeles California

**Keywords:** conversation analysis, emergency medicine, medical communication, online commentary, physical examination, primary care

## Abstract

This paper describes a communication practice called “online commentary” that is in widespread use in primary care in the USA. Online commentary is talk by a clinician that describes what he or she is finding in the course of the physical examination of the patient. The paper reviews the primary features of online commentary, with a special focus on its role in forecasting the likely results of the physical examination during the examination itself. It also describes patient outcomes that are associated with this use. It then uses data from an emergency room in the western USA to extend the notion of online commentary from primary care to the emergency setting. It proposes that online commentary facilitates effective teamwork by forecasting next actions, allowing members of the emergency team to anticipate probable next steps in the investigation and treatment of patient injuries.

This paper reports on a communication practice that is in widespread use in US primary care settings during the physical examination of the patient. This practice is called “online commentary”. Online commentary is talk that describes what the physician is seeing, feeling, hearing, or otherwise encountering during the physical examination of the patient.^1^ Online commentary use in primary care is common. In a study of communications in a pediatric setting in which children were presenting with Acute Respiratory Tract Infections (ARTI), over 70% of physicians were found to make online comments during the physical examination.^2^ Our study used conversation analysis, a sociological method involving the detailed analysis of language use in medicine, allied with quantitative methods, to research this communication behavior.

Medical textbooks of physical examination are divided about the clinical value of online commentary. For example, Billings and Stoeckle^3^ recommend that “when the examination is normal, let the patient know. Everyone appreciates this good news, both during the examination and at the end of the consultation.” Zoppi^4^ agrees that talk during the examination of the patient can serve a reassuring function: “physical findings should be described to the patient, who otherwise may misinterpret a squint and silence as cues that something… is horribly wrong.” A more cautious note is sounded by Bates *et al*.^5^ who comment that while such remarks can increase “both the credibility and the conviction of the clinician's advice or reassurance”, they also have drawbacks: “A steady series of reassuring comments, however, presents at least one potential problem: what to say when you find an unexpected abnormality. You may wish you had maintained judicious silence earlier.” Using similar reasoning, Swartz^6^ proposes a strongly negative view of this practice: “The examiner should always refrain from comments such as ‘That's good’ or ‘That's normal’ or ‘That's fine’ in reference to any part of the examination. Although this is initially reassuring to the patient, if the examiner fails to make such a statement during another part of the examination, the patient will automatically assume that there is something wrong or abnormal.”

Our investigations of online commentary focused on its function in forecasting diagnostic and treatment outcomes in pediatric consultations.^1^, ^2^ Our research provided the first statistical evidence indicating that online commentary could reduce patient and parent expectations that antibiotics would be prescribed, as well as actual levels of inappropriate prescribing associated with the pressure of these expectations.^7^ We illustrate this process through Examples 1–4 below.

In a case involving an 11‐year‐old child presenting with left side ear pain who was described by her mother as suffering from an “ear infection,” an indicator that the mother was looking for an antibiotic prescription,^7^ the exchange detailed in Table 1 occurred while the pediatrician was checking the child's ears.

**Table 1:** Example 1 of online commentary: exchange between an 11‐year‐old female patient (PAT), the patient's mother (MO), and the attending physician (DOC)

1	DOC:		Which ear's hurting or are both of them hurting.
2			(0.2)
3	PAT:		Thuh left one,
4	DOC:	‐>	°Okay.° This one looks perfect,.hh ((Examining the left ear))
5	MO?:		(U[h:.???)
6	DOC:	‐>	[An:d thuh right one, also loo:ks, (0.2) even more
7		‐>	perfect. ((Examining the right ear))
8	PA?:		()
9	DOC:		Does it hurt when I move your ears like that?
10			(0.5)
11	PAT:		No:.

In this case, while actually examining each ear, each of the physician's comments (lines 4, 6, and 7) points to an absence of medical signs. These comments carry the implication that the child may not be suffering from the “ear infection” that the mother named when presenting the child's chief complaint. In turn, these comments may also suggest that the physician is not encountering a condition that can be appropriately treated with antibiotics. For while certain forms of *otitis media* are appropriately treated in this way, an asymptomatic child certainly should not be treated with antibiotics. Moreover, the overt reporting of absent signs not only begins to build a case against inappropriate prescribing, it also starts to publicly position the physician as disinclined to do so, and forecasts to the parent that an antibiotic prescription is not likely to be the treatment outcome of the visit.

Cases like this one alerted us to the possibility that online commentary might be a useful communication tool with which physicians can resist perceived parental pressure^8^ to prescribe antibiotics inappropriately for ARTIs. In the USA, this pressure can arise from a variety of sources. Anticipating a bacterial diagnosis, parents may be looking for a “quick fix” to ease their child's suffering, to alleviate sleepless nights, to allow their children to attend schools and enable them to go to work, or to allow scheduled airline trips or children's parties to go ahead as planned. Concerned with legitimizing the visit to the pediatrician, parents may feel that only prescription medications justify the visit or make it worthwhile.^1^, ^9^, ^10^

In a context where inappropriate prescribing in pediatric contexts is widespread^11^, ^12^, ^13^ and a known risk factor is the development of antibiotic resistant microbes,^14^, ^15^, ^16^, ^17^ online commentary appeared worthy of further investigation.

## What is online commentary?

We describe online commentary in terms of several co‐occurring characteristics.

1. It is produced as subordinate to the activities of the physical examination, occurring simultaneously with an act of examination (for example, while the physician is undertaking an otoscopic examination of a patient's ear), or between successive elements in an examination comprising multiple actions (for example, examination of a patient's ears, throat, and sinuses).
2. It is mainly used to report on signs that were absent, or present but mild, and which are treated as non‐problematic by the physician. Online commentary that describes absent signs often uses mitigating “evidential” formulations, for example, “I don't see any fluid.” Evidential formulations involve the use of verbs like “see”, “feel”, “smell”, and “hear” that make reference to the perceptual evidence from which observations come.^18^ They are a way of downgrading claims. The claim “I don't see any fluid” is not as strong as “There isn't any fluid” because it leaves open the possibility of there being fluid which is unseen. Online commentary that describes signs that are present but mild ordinarily takes the form of simple assertions, normally using terms that are downgraded or qualified, for example, “That's a *little bit* red back there,” or “There may be a *little bit* of lymph node swelling on this side compared to the other side”.
3. In a minority of cases, physicians use online commentary to describe problematic signs. These would take the form of simple assertions, for example, “That's quite inflamed.”
4. Online comments addressing both present and absent signs take two primary formats: (i) as reports of observations, such as “I don't see any fluid” or “Little bit red”, (ii) as assessments of what is observed, such as “Your ears look good” or “This one looks perfect”. In the report format, the physician does not formulate an overt evaluation about the significance of an observation for the patient's health status, leaving it to the patient to draw their own conclusions about it. In the assessment format, conclusions are overtly drawn.
5. The subordination of online commentary to the activities of physical examination emerges in the fact that it is rarely overtly addressed to patients or directly acknowledged by them. For example, online comments are typically delivered without gazing at the patient, which is a primary index of talk that is directly addressed to a recipient.^19^, ^20^ Online comments are also rarely responded to by patients. Additionally, the patient may often be physically unable to respond during an examination (for example an otoscopic examination of the throat or sinuses). Moreover, even when patients are physically able to respond, they typically lack access to what the physician is observing, as frequently the examinations involve tools (e.g. otoscopes, stethoscopes), and their interpretation normally requires medical expertise.^21^ Thus, the preconditions for response—shared access to the object under evaluation^22^—are absent.

## How does online commentary work in pediatric ARTI cases?

In this section, we use a single case to illustrate the workings of online commentary in moving towards a “no problem, no prescription” treatment recommendation. Our case is a continuation of Case 1 above, in which the parent presented her daughter as having an “ear infection”. As we have seen, the physician's use of online commentary began the process of blocking that possibility, and subsequently the mother began to entertain an alternative symptom “sore throat pain,” which the physician pursues in Table 2.

**Table 2:** Example 2 of online commentary: exchange between an 11‐year‐old female patient (PAT), the patient's mother (MOM), and the attending physician (DOC)

1	DOC:		Uh: let's see. Say ah:,
2			(0.5)
3	PAT:		(uh_??)
4			(1.0)
5	DOC:	‐>	That's uh little bit red back there,
6			(0.2)
7	DOC:	‐>	I don't see anything: (0.4) °Yeah.° Very good. Thank you.
8	MOM:		Huh h[uh huh (.hh)
9	DOC:	‐>	[I don't see anything (.) that looks infected.
10	MOM:		Reall[y, °Okay.°
11	DOC:	‐>	[Uh: in thuh sense that we're: looking at
12		‐>	bacterial, strep throat kinda thing(s).
13	DOC:		.h[h
14	MOM:		[O[kay.
15	DOC:		[Lemme listen to ya.
16			(1.5)
17	MOM:		Could it be [from allergie:s,
18	DOC:		[Take uh deep breath, Sit up straight?,

Here, the physician again uses online commentary in an implicit rebuttal of the mother's suggestion. However, at line 5, he begins by identifying a sign that is present but mild. This online comment could validate the child's complaint of sore throat pain, and hence the mother's decision to make the medical visit. Subsequently, having completed the examination and while preparing to listen to the child's lungs, he produces a more comprehensive online report, which is also evidentially formulated: “I don't see anything (.) that looks infected” (line 9). Subsequent to the mother's possibly resistant “Really” (line 10), he qualifies his previous assessment (lines 11–12), in a way that both allows that the child may still have some kind of infection, while eliminating the prospect of a bacterial infection and, by implication, the prospect of antibiotic treatment.

The mother's response to this outcome at line 17 is to maintain her position that her daughter has a medically treatable problem, by raising the prospect of a further condition, allergies. Insofar as this inquires into a different diagnosis of the problem, it displays her acquiescence to the physician's rejection of “strep throat” as a diagnosis.

After an uneventful lung examination, the pediatrician moves to examine the girl's lymph nodes. This examination is shown in Table 3.

**Table 3:** Example 3 of online commentary: exchange between the mother of an 11‐year‐old female patient (MOM) and the attending physician (DOC)

1	DOC:		Does it hurt when you breathe in deep like that?
2			(1.4) ((Patient shakes head))
3	DOC:		No:?
4			(0.2)
5	DOC:		How ‘bout‐ under your chinny chin chin.<°Let's see.°
6			(1.5)
7	DOC:	‐>	No(w) there may be uh little bit of lymph no:de swelling
8		‐>	on this side com[pared to the other side,
9	MO?:		[(Yeah.)
10			(.)
11	DOC:	‐>	On thuh [left side,
12	MOM:		[Oh:: okay.

The outcome of this examination offers some further support for the existence of a medical problem, albeit slightly downgraded with the modal “may” (line 7). The physician's identification of “lymph node swelling” is presented online, and gives implicit support to the patient's claim that she has experienced pain primarily on the left side.

Subsequently the physician makes this explicit in his diagnosis, which he begins with the upshot formulating “so” at line 1 (Table 4).

**Table 4:** Example 4 of online commentary: exchange between the mother of an 11‐year‐old female patient (MOM) and the attending physician (DOC)

1	DOC:	.hh So: it would loo:k hh like she is:=uhm (.) prob'ly
2		fighting some (.) viral: upper respiratory kinda stuff,
3		.hh More on thuh left than on thuh right, which
4		c[an account for some pain maybe,
5	MOM:	[Okay.
6		….
7		…. ((13 lines of ear compliment sequence removed))
8		….
9	DOC:	Uh:‐ I would tell you though I don't hhh (.) I don't see
10		anything that requires like antibio:tics er anythi:ng,
11		but certainly sympto[matic treatment might be in order,
12	MOM:	[Mm.
13	DOC:	.hh
14	MOM:	O[kay.
15	DOC:	[Uhm: anything from vaporizers tuh maybe some chloraseptic
16		kinda stuff for thuh [throat, lozenges might be better,
17	MOM:	[Oh:. Okay.

The first part of this diagnostic evaluation (Table 4, lines 1–4) builds from his most recent online comment (in Table 3), and gives some support for the mother and daughter's decision to seek medical care for the condition. The second part (Table 4, lines 9–11) builds on his adverse online commentary (Tables 1, 2) and explicitly rejects antibiotic treatment in favor of symptomatic, non‐prescription remedies. In particular, he builds the recommendation as contrastive with the notion that viral conditions require antibiotic treatment, and hence contrastive with any position the mother might hold in favor of antibiotic treatment, without contradicting his earlier evaluation (Table 4, lines 1–4) that the child is nonetheless sick. His use of the evidential formulation (“I don't see anything…”) revives the relevance of the observations reported in his earlier online comments, and re‐invokes their significance as evidence for the position he is currently taking. Across this sequence, the mother responds to both the supportive and adverse aspects of the diagnostic evaluation with an acknowledgement token “okay” which accepts the physician's evaluation. At line 17, this acceptance becomes more marked with the addition of “oh”.^23^ Subsequent to this, the mother discusses the merits of several commercial remedies in a cordial way, and without contesting any aspect of the physician's conclusions.

### What are the underlying factors that allow the physician to achieve this outcome through online commentary?

1. He has used online commentary to validate that the patient has some legitimate signs of illness (redness in the throat and swollen lymph nodes), and suggested—in line with the patient's claims—that these signs are more marked on the left than the right.
2. He has reassured the parent that the signs are mild.
3. He has consistently shaped the parent's expectations away from a prescription treatment recommendation, while simultaneously committing himself to that outcome.
4. His diagnoses were built from, and to some extent recapitulated, the earlier online comments.

In achieving this objective, the physician also draws on what the sociologist Paul Starr^24^ calls the cultural authority of medicine. Physicians are trained to look at ears, throats, and sinuses, and they are professionally authorized to evaluate the state of these organs for a living. From the layman's point of view, their observations define the state of these areas. They are culturally empowered to offer definitive conclusions. Patients are correspondingly not normatively entitled to contradict them. This is reflected in studies that examine patients’ argumentative responses to physicians’ diagnoses. For example, Peräkylä^25^ found that patients never contradicted the evidence that physicians describe, even when they disagreed with their diagnoses.

Moreover, because online commentary is delivered while the examination is still in progress, they are not (at least, not yet) conclusive. They are staging posts on the way to a conclusion. Because they are observations that build up evidence incrementally, rather than asserting it conclusively, they are not to be treated, in themselves, as objects for discussion or disagreement.

Finally, these comments often address areas of the patient's body—for example, the ears and sinuses—that the patient is rarely in a position to see, and which even the parent of a child would have difficulty in observing without an otoscope. Physicians have what we might term an epistemic “ecological advantage” in examining these areas: these parts of human anatomy are easily visible to physicians, but quite difficult for patients or others to examine.

For all these reasons, the physician effectively builds a case against antibiotic treatment, piece by piece, and in a fashion that is extremely difficult for the parent to contradict. Whether consciously or not, by reporting each observation as it is made, the physician progressively builds a more or less unanswerable case for the diagnostic conclusions he ultimately asserts.

## Pattern of online commentary use

As previously noted, most physicians who use online commentary in the context of pediatric ARTIs primarily deploy what may be termed “no problem, no treatment” online observations. However, not all do so. We have already noted that physicians may produce “problem” online commentary, for example, “That looks infected.” We have also noted that online commentary is incremental. It follows that an incremental sequence of “no problem” online comments can be interrupted and overturned at any point by a single announcement of a “problem” online comment.

In examining the incidence of online commentary, we therefore compare three classes of cases in which the physician: (i) did not engage in online commentary, (ii) exclusively used “no problem” online commentary, (iii) used at least one “problem” online comment. We divided our ARTI cases into those where a bacterial diagnosis was assigned, and those where a viral diagnosis was the outcome. Among the bacterial cases, physicians’ use of online commentary showed no significant differences when they believed that the parents expected, or did not expect, an antibiotic prescription. Among the viral cases, however, a significant difference emerged. Once again, similar rates of “no problem” online commentary were evident regardless of whether the physician believed that the parent expected, or did not expect, an antibiotic. However, in the viral cases, physicians were considerably more likely to use “problem” online commentary when they thought the parent expected an antibiotic prescription.^2^ In these cases, the possibility arises that online commentary was used to align with perceived parent demand, and indeed, inappropriate prescribing was more common when “problem” online commentary was used.^2^

It may be concluded then that online commentary is both a tool for shaping expectations and forecasting outcomes, but also may be used as a means to forecast acquiescence to perceived patient expectations. Either way it is a powerful tool for forecasting next steps, shaping expectations, and influencing outcomes. How then does online commentary function in the emergency room (ER) context?

## Online commentary in the ER

When considering online commentary in the ER, it is of course its role in alerting others and forecasting next steps that is central. As Maynard notes in his paper in this issue, dictionary definitions of forecasting suggest two meanings: (i) “to serve as an advance indication” of something to come, (ii) to “estimate or calculate in advance.” Both meanings are relevant in the ER context. Consider the following protocol from the ER in a level 1 trauma hospital in a large city in the western USA [This protocol is based on ethnographic observations, and comes from Goldstein (2015)^26^].

This ER team consists of doctors, nurses, technicians, radiologists, and support staff from both the Emergency Department and the Department of Surgery. The size of the team ranges from 12 to 30 clinicians, and includes an attending physician, an Emergency Department chief resident, several nurses including an registered nurse scribe, a radiologist, and two residents positioned at the head and foot of the patient. In the following case, the patient has just arrived at the ER. He has crashed his car into a pole, and has briefly lost consciousness.

The team follows the Advanced Trauma Life Support Program for Doctors (ATLS) protocol,^27^ and the initial observations are in line with the “airway, breathing, circulation” elements of the primary survey mandated by the protocol (Table 5).

**Table 5:** Example 5 of online commentary: exchange between emergency room staff on arrival of a patient who has been involved in a motor vehicle accident

1	HEAD:	Airway intact. Lungs equal.
2	CHIEF:	No chest flail. No pelvic instability.
3	RN SCRIBE:	Thanks.
4	RADIOLOGIST:	X‐RAY. X‐RAY. Chest X‐ray.
5		((The team pauses for 2 s))
6	RADIOLOGIST:	OK clear.
7	HEAD:	Breath sounds equal and bilateral.
8	CHIEF:	Moving all extremities.
9		((Attending on right of the patient is ultrasounding abdomen.
10		The tech cuts off the patient's clothes))
11	HEAD:	Small abrasion on nose uh sorry the uh left cheek.
12		((HEAD asks medical history questions to the patient))
13	FOOT:	Does he have any pain?
14	HEAD:	Did you lose consciousness?
15	FD MEDIC:	Per witness, he lost consciousness for 3 to 4 min. They also
16		said he wasn't breathing.
17	HEAD:	A and O times 3.
18	HEAD:	No C‐spine tenderness.
20	CHIEF:	Pelvis stable.
21	TECH:	100 on room air.
22	NURSE:	First HemoCue 16.0.

The Emergency Room team includes an attending physician, an Emergency Department chief resident (CHIEF), several nurses including an registered nurse scribe (RN SCRIBE), a radiologist, and two residents positioned at the head (HEAD) and foot (FOOT) of the patient. Fire Department Medic (FD MEDIC), and a medical technician (TECH).

Most of the remarks in this transcript consist of online commentary (lines 1, 2, 6, 7, 8, 11, 17–22). Most of them announce vital signs concerning the patient's airway (line 1), breathing and circulation (lines 1, 2 and 7), lateralization signs (line 8), injuries (line 11), alertness (line 17), condition of spinal cord (line 18), blood oxygenation (line 21), and red blood cell count (line 22). Together this succession of online comments permits the team to construct and orient to a shared understanding of the patient's condition. Thus, by line 13, the clinician at the foot of the bed is ready to raise the question of pain, and the clinician at the head of the bed is able to focus on the patient's possible loss of consciousness (line 14), and current level of alertness (line 17). In organizing a shared understanding of the patient's condition, this body of online commentary also and simultaneously unifies the team of clinicians in terms of shared tasks and shared clinical focus. It also permits an organizational structure in which the attending physician can maintain a “hands off” overall view of the patient and the progress of the treatment. As Goldstein notes:

“While the chief resident positions herself at the patient's right hip, the attending physician (the ultimate authority in the room) stands back a step or two looking at monitors and over the shoulders of the clinicians at the patient's bedside….

While the team announces their findings in the general order prescribed by the ATLS, the attending is not even looking at the patient. His visual attention is focused on the monitor of the ultrasound console facing away from the patient's body. Through the announcements of the team's findings, however, he is able to look through the “eyes” of the trauma team … without returning his gaze to the conscious patient; the attending can see without being seen.”^26^

Overall, as Goldstein also notes, the procedure creates a horizontal division of labor among the team members, while also creating a control structure through which the attending can intervene if necessary.

## Conclusion

This paper has advanced two roles for online commentary. In communicating with patients, it suggests that patient expectations about diagnoses and treatment recommendations can be structured by online comments during the physical examination. In the multidisciplinary teamwork context of the ER, it suggests that online commentary can create a shared focus on a variety of vital signs, and project next actions based on the ATLS protocol. In the flow of teamwork created through online commentary, the team itself is consolidated through shared understandings and shared tasks. Additionally, online commentary creates a rich informational context in which team leaders who, ideally, remain at a “monitoring distance” from the direct action,^28^ are fully informed in the same way as other team members, but can maintain the kind of overall view that is required to direct, or redirect, the work of the team.

The common factor in these two quite different implementations of online commentary is its role in forecasting what is to come. Simple observations during the physical exam convey to patients what the likely diagnosis and treatment plan will be. They convey this indirectly, through inference. Similarly, in the ER, online commentary on vital signs alerts team members indirectly about which tasks will emerge next, and which can be ruled out. In both contexts, the forecasting enabled by online commentary contributes to effective medical care and positive patient outcomes.

## Conflict of interest

None.
